# Train Axlebox Bearing Fault Diagnosis Based on MSC–SGMD

**DOI:** 10.3390/s24010254

**Published:** 2023-12-31

**Authors:** Yongliang Bai, Hai Xue, Jiangtao Chen

**Affiliations:** School of Mechanical Engineering, Lanzhou Jiaotong University, Lanzhou 730070, China; yongliangbai@126.com (Y.B.); 11220334@stu.lzjtu.edu.cn (J.C.)

**Keywords:** spectral coherence, symplectic geometry mode decomposition, axlebox bearing compound faults

## Abstract

Train axlebox bearings are subject to harsh service conditions, and the difficulty of diagnosing compound faults has brought greater challenges to the maintenance of high–quality train performance. In this paper, based on the traditional symplectic geometry mode decomposition (SGMD) algorithm, a maximum spectral coherence signal reconstruction algorithm is proposed to extract the intrinsic connection between the SGMD components with the help of the frequency domain coherence idea and reconstruct the key signal components so as to effectively improve the extraction of composite fault features of axlebox bearings under different speed conditions. Firstly, based on the traditional SGMD algorithm, the vibration signal of the axle box is decomposed to extract its symplectic geometry components (SGCs). Secondly, the spectral coherence coefficient between the SGCs is calculated, and the signal in which the maximum value is located is taken as the key component for the additive reconstruction Finally, the envelope spectrum is used to extract the reconstructed signal fault features. The inner race, outer race, and compound bearing failure vibration signal acquisition under different speed conditions were carried out on the equal scale axlebox bearing failure simulation test bench, and the effectiveness of the proposed algorithm was verified based on the axlebox vertical acceleration signal.

## 1. Introduction

Accompanied by the growing number of rail trains and operating mileage, the safe operation and maintenance of these vehicles have become a crucial focus in the rail transportation industry. As one of the most critical rotating parts in the vehicle’s traveling section, the research of reliable maintenance and fault diagnosis algorithms for axlebox bearings can significantly impact the intelligent and green operation of rail trains [1,2]. However, the harsh service conditions of rail train axlebox bearings, including wheel–rail friction wear, time–varying coupling wheel–rail impact excitation, non–smooth load excitation of the vehicle body, and non–linear speed excitation of the bogie can lead to various localized failures [3,4,5,6,7]. Statistical data reveal that bearing faults in rail vehicles are primarily concentrated in the cage, inner and outer races, and rolling elements, with a higher incidence of faults occurring in high–speed, heavy–duty operating conditions [8,9,10,11]. Therefore, accurate diagnosis of single and compound faults of axlebox bearings is crucial to ensure the healthy service state of these components and maintain the reliable operation of rail trains.

However, due to the challenging operating environment of train axlebox bearings, their fault vibration signals are intricate, exhibiting serious frequency modulation phenomena, and the increased noise from the complex conditions hinders effective feature extraction. To address this problem, scholars have proposed various modal decomposition methods, including classical empirical mode decomposition (EMD) [12], variational mode decomposition (VMD) [13,14,15], local mean decomposition (LMD) [16], and others. These methods are widely used in the decomposition and reconstruction process of bearing fault vibration signals. It is worth emphasizing that these methods aim to hierarchically peel off the original signal based on specific criteria and reconstruct the separated components, which are then used to characterize the signal within a specific range. Building upon these modal decomposition methods, some scholars have developed an algorithm called symplectic geometric mode decomposition (SGMD) [17,18]. The core of this algorithm is the symplectic matrix similarity transform, which solves the eigenvalue problem for the target matrix and reconstructs the SGC using its corresponding eigenvectors. This denoises the complex signals and enables adaptive decomposition, resulting in a number of distinct single–component components of the SGC. Subsequent research has focused on refining the SGMD algorithm for faulty vibration signal decomposition and applying intelligent diagnosis networks [19,20,21].

Our method, called MSC–SGMD, utilizes the reconstruction principle of normalized mean square error (NMSE) after decomposing the signal into individual components. This principle determines convergence by iteratively calculating the time series correlation coefficient between each component and the first component, which is effective for time series vibration signals [22,23]. However, this method does not yield significant separation results for composite fault vibration signals in train axleboxes. The large number of components in train axleboxes and various interferences such as serpentine motion and wheel–rail excitation result in strong similarity between the time series of each component. Consequently, using the aforementioned method to calculate the time series correlation coefficient has certain limitations. It often leads to a small number of reconstructed signals and a centralized reconstructed band, making it difficult to accurately separate vibration bands associated with composite faults. Therefore, it is necessary to propose a signal reconstruction principle based on spectral coherence that takes into account the operating frequency range characteristics of train axlebox bearings. This would ensure that there is no over–concentration of fault frequency bands during reconstruction of compound fault signals in train axleboxes.

This paper proposes a novel approach for separating compound fault diagnostic signals from train axlebox bearing vibrations based on the characteristics of the frequency band range of the compound fault vibration. Our method, called the maximum spectral coherence symplectic geometric mode decomposition (MSC–SGMD) component reconstruction algorithm, leverages maximum spectral coherence to enhance the performance of SGMD in separating the compound fault diagnostic signals. By combining the mechanical vibration characteristics of the train axlebox, we extract the corresponding compound fault vibration characteristics from the newly reconstructed signals, enabling effective diagnosis of compound faults. The algorithm presented in this paper offers theoretical support for the decomposition and reconstruction of vibration signals associated with bearing faults. Simultaneously, it serves as a valuable algorithmic reference for the development of guidelines for reconstructing other single components. Employing this algorithm in the analysis of vibration signals from wheelset bearings significantly enhances the precision of diagnosing faults in axlebox bearings. This timely and accurate diagnosis ensures the optimal performance of axlebox bearings in service, contributing effectively to the safety and economic efficiency of train operations.

## 2. MSC–SGMD Algorithm

### 2.1. Traditional NMSE–SGMD

The primary concept of the conventional NMSE–SGMD is to calculate the variation of the temporal signal’s octant matrix and solve for eigenvalues using the Hamilton matrix. Subsequently, it iteratively computes the normalized mean squared error between components. When the coefficient calculation result surpasses a threshold (typically 0.95), it signifies that the reconstructed part is an independent component. For discrete vibration signals $x\left(i\right)=\left(x_{1},x_{2},\cdots ,x_{n}\right)$, the decomposition process based on SGMD is as follows: 

#### 2.1.1. Phase Space Reconstruction

The multi–dimensional signal is reconstructed based on Takens embedding, and the trajectory matrix X is constructed by using one–dimensional input time series delay topology algorithm.

(1) $$X=\left[\begin{array}{cccc} x_{1} & x_{1+\tau } & \cdots & x_{1+\left(d-1\right)\tau }\\ \vdots & \vdots & & \vdots \\ x_{m} & x_{m+\tau } & \cdots & x_{m+\left(d-1\right)\tau } \end{array}\right]$$

where d is the embedding dimension, τ is the delay time, and m=n−(d−1)τ. Here, the embedding dimension determines the final number of SGCs, and its value criterion is as follows.

(2) $$d=\{\begin{array}{cc} round\left(n/3\right) & f_{\max}/F_{s}<10^{-3}\\ 1.2\times \left(F_{s}/f_{max}()\right) & f_{\max}/F_{s}\geq 10^{-3} \end{array}$$

#### 2.1.2. Symplectic Geometric Matrix Transformation

The objective is to obtain the Hamilton matrix, which can extract the time–displacement similarity of its own sequence by utilizing the trajectory matrix. This enables the calculation of the covariance symmetric matrix.

(3) $$A=X^{T}X$$

Then, the Hamilton matrix M is represented based on the following matrix.

(4) $$M=\left[\begin{array}{cc} A & 0\\ 0 & -A^{T} \end{array}\right]$$

After obtaining the Hamilton matrix, it is desirable to square the matrix ($N=M^{2}$) to satisfy the Hamilton matrix property. It can be obtained from the above content and satisfy the characteristics of Hamilton matrix, so it can realize the symplectic orthogonal matrix Q representation as follows.

(5) $$Q^{T}NQ=\left[\begin{array}{cc} B & R\\ 0 & B^{T} \end{array}\right]$$

#### 2.1.3. Diagonal Averaging

The dimension of the original single component obtained from the above steps is $Z_{i}$, and the component combination can be further arranged. By definition, 1≤i≤d,1≤j≤m can be obtained. Therefore, the corresponding diagonal averaging transformation matrix is expressed as follows.

(6) $$y_{k}=\{\begin{array}{cc} \frac{1}{k}\sum_{p=1}^{k}z_{p,k-p+1}^{*} & 1⩽k<d^{*}\\ \frac{1}{d^{*}}\sum_{p=1}^{d^{*}}z_{p,k-p+1}^{*} & d^{*}<k⩽m^{*}\\ \frac{1}{n-k+1}\sum_{p=k-m^{*}+1}^{n-m^{*}+1}z_{p,k-p+1}^{*} & m^{*}<k⩽n \end{array}$$

Furthermore, based on the diagonal averaging operation, a single component combination $Y=Y_{1}+Y_{2}+\cdots +Y_{d}$ can be obtained. 

#### 2.1.4. NMSE Reconstruction

In the signal component group obtained using the decomposition algorithm, the primary component is positioned at the beginning of the matrix element. Hence, the first original single–component signals are used as a basis for feature analysis and comparison with the remaining components. We select and reconstruct components with high similarity to obtain the first signal component. Subsequently, by utilizing these reconstructed components, we derive a residual matrix which allows us to calculate a residual signal through summation. To assess accuracy, we calculate normalized mean square error (NMSE) by comparing the residual term with the initial signal.

(7) $$NMSE=\frac{\sum_{e=1}^{n}g^{h}\left(e\right)}{\sum_{e=1}^{n}x\left(e\right)}$$

where h represents the number of iterations in the loop. When NMSE<th,th=0.01, the corresponding iterative algorithm is terminated. Otherwise, the initial matrix repetition operation based on the residual matrix is performed until the cutoff condition is met.

(8) $$x\left(n\right)=\sum_{h=1}^{N}SGC^{h}\left(n\right)+g^{\left(N+1\right)}\left(n\right)$$

### 2.2. Maximum Spectral Coherence

The spectral coherence calculation extracts the phase and spectral magnitude correlation between the quantities being computed based on temporal correlation [24,25,26]. By calculating the cosine of the angle of the input vector in the high–dimensional space, each frequency point’s spectral value is treated as a random variable, and the correlation between the two random variables is calculated (the cosine of the angle of the vector). In summary, spectral coherence primarily expresses the similarity of two time series in the frequency domain. For time series signals, the spectral coherence coefficient is calculated as follows:

(9) $$C_{xy}\left(f\right)=\frac{{\left|G_{xy}\left(f\right)\right|}^{2}}{G_{xx}\left(f\right)G_{yy}\left(f\right)}$$

The cross–spectral density of x(t) versus y(t) is denoted as $C_{xy}\left(f\right)$. By expanding this expression, we can separate the signal self–power spectrum and mutual power spectrum. This expansion yields:

(10) $$C_{xy}\left(f\right)=\frac{{\left|E\left\{X^{*}\left(f\right)Y\left(f\right)\right\}\right|}^{2}}{E\left\{X^{*}\left(f\right)X\left(f\right)\right\}E\left\{Y^{*}\left(f\right)Y\left(f\right)\right\}}=\frac{{\left|E\left\{X^{*}\left(f\right)Y\left(f\right)\right\}\right|}^{2}}{E\left\{|X\left(f\right){|}^{2}\right\}E\left\{|Y\left(f\right){|}^{2}\right\}}$$

In the actual signal processing process, the spectral window is utilized to perform the calculation, and the mean value in the window is used to estimate the corresponding expectation, respectively, i.e., there is a signal, and the spectral coherence between is calculated as follows.

(11) $$C_{xy}\left(f\right)=\frac{\frac{1}{N^{2}}|\sum_{i=1}^{N}X_{i}^{*}\left(f\right)Y_{i}\left(f\right){|}^{2}}{\frac{1}{N^{2}}\left(\sum_{i=1}^{N}|X_{i}\left(f\right){|}^{2}\right)(\sum_{i=1}^{N}|Y_{i}\left(f\right){|}^{2})}={\left|\frac{\sum_{i=1}^{N}|X_{i}^{*}\left(f\right)Y_{i}\left(f\right)}{\left(\sum_{i=1}^{N}|X_{i}\left(f\right){|}^{2}\right)(\sum_{i=1}^{N}|Y_{i}\left(f\right){|}^{2})}\right|}^{2}$$

Furthermore, the spectral coherence coefficients are calculated to allow qualitative and quantitative calculation of the spectral coherence between the components.

### 2.3. MSC–SGMD

Furthermore, the SGMD reconstruction algorithm based on the maximum spectral coherence coefficient can be implemented by combining it with the aforementioned algorithm principle. By applying the traditional SGMD algorithm to a vibration signal S(t), we can obtain its corresponding single–component signals (SGCs).

(12) $${SGCs\left(i\right)|}_{i=1}^{N}=sgmd\left(St\right)=\sum_{h=1}^{N}SGC^{h}\left(n\right)+g^{\left(N+1\right)}\left(n\right)$$

Moreover, once the −*SGCs* have been obtained, they can be filtered and reconstructed by applying the aforementioned principle of maximum spectral coherence (MSC). By utilizing the ${SGCs\left(i\right)|}_{i=1}^{N}$ as input signals, an iterative computation is carried out to derive the matrix *M*, which represents the coefficient of spectral coherence.

(13) $$M_{NN=}C_{\left(SGCs\left(p\right)\right)\left(SGCs\left(q\right)\right)}={\left|\frac{\sum_{i=1}^{N}|SGCs{\left(p\right)}_{i}^{*}\left(f\right)SGCs{\left(q\right)}_{i}\left(f\right)}{\left(\sum_{i=1}^{N}|SGCs{\left(p\right)}_{i}\left(f\right){|}^{2}\right)(\sum_{i=1}^{N}|SGCs{\left(q\right)}_{i}\left(f\right){|}^{2})}\right|}^{2}$$

where *p*, *q* = 1:*N*.

The matrix representation of Equation (13) readily reveals that the M–matrix is symmetric, with its spectral coherence coefficients exhibiting symmetry along the main diagonal.

The new MSC–SGMD–based reconstructed signal Sn can be obtained by selecting the maximum value from the M matrix and taking the coordinates of its location corresponding to the components of the SGCs to combine them.

(14) $$S_{n}=\sum SGCs\left(l,m\right){|}_{l,m\in Max\left(M\left(l,m\right)\right)}$$

As a result, the reconstructed signal Sn based on MSC–SGMD can be obtained. Subsequent analysis of its envelope spectrum enables the extraction of compound fault features.

## 3. Experimental Verification of Equal Proportions

### 3.1. Test Bench Introduction

**Figure 1:**
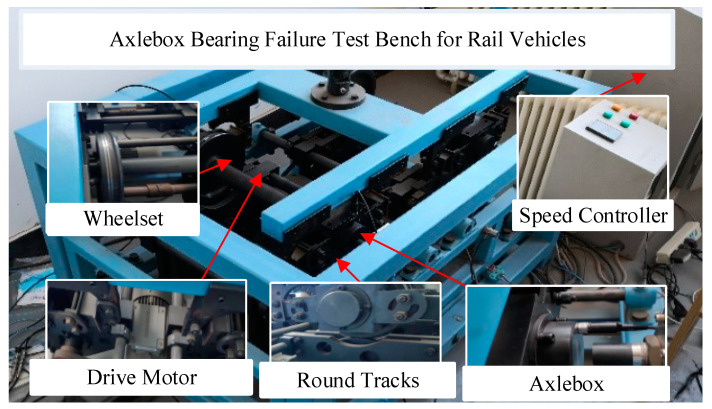
Scaled test bench.

To evaluate the performance of the MSC–SGMD signal–processing algorithm proposed in this paper, we conducted experiments in a laboratory setting using a scaled–down rail vehicle bogie. We compared the diagnostic accuracy of faults in bearings using the acquired vibration signals and the proposed algorithm. The experimental setup is depicted in Figure 1.

**Figure 2:**
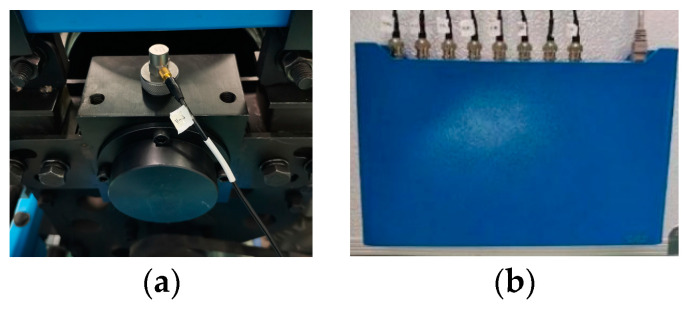
(**a**) Vertical IEPE accelerometer. (**b**) Dynamic signal acquisition system.

The test bench features a 1:5 scale train bogie structure, comprising wheel pairs, speed controllers, drive motors, circular rails, axleboxes, and other components. The propulsion motor is regulated by the speed controller to supply energy for the circular track, inducing the rotation of the wheel pair through the friction generated between the tread and the wheel pair. Concurrently, the test bench features four axle boxes dedicated to the wheelsets, each outfitted with four sets of double–row tapered roller bearings to facilitate the rotation of the wheelsets. Additionally, the test bench is equipped with an Integrated Electronics Piezo–Electric (IEPE) acceleration sensor to measure the axlebox vertical acceleration signal. Utilizing the high–performance dynamic signal collector, the vertical vibration acceleration signals from the axlebox bearings can be efficiently captured, providing a realistic representation of the vibration status of the wheelset’s axlebox bearings. The corresponding IEPE acceleration sensor and dynamic signal acquisition system are depicted in Figure 2.

Through simulating faults in the axle box bearing and operating it at various speeds, different fault vibration signals can be obtained. This paper employs the wire–cutting method to simulate bearing faults in the inner, outer races and compound faults of the axle box bearing, as shown in Table 1.

**Figure 3:**
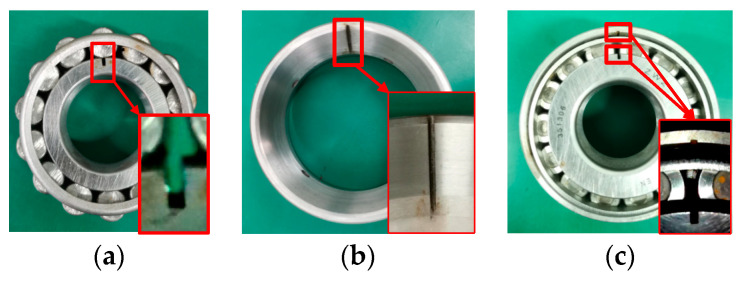
(**a**) Case 1: Inner race fault. (**b**) Case 2: Outer race fault. (**c**) Case 3: Compound fault.

The working condition was subjected to fault simulation by machining 1 mm wide grooves on the working surface of the inner and outer races of the axlebox bearing of the wheelset using wire cutting. Furthermore, the train bearing fault simulation was conducted by setting the line speeds at 10 km/h for speed 1, 30 km/h for speed 2, and 50 km/h for speed 3. The fault simulation bearings used for this purpose are depicted in Figure 3.

The bearing type is 351306 double–row tapered roller bearings. The ball pass frequency outer race (*F_BPFO_*) and ball pass frequency inner race (*F_BPFI_*) of this bearing should be calculated as

(15) $$\{\begin{array}{c} F_{BPFI}=f_{r}\times 8.322\\ F_{BPFO}=f_{r}\times 5.678 \end{array}$$

where *f_r_* is the rotation frequency of the wheelset. In all cases, the sampling frequency Fs was set to 20,000 Hz in order to meet the Nyquist sampling requirement and to obtain a good frequency spectrum resolution. The 351306 double–row tapered roller bearing has an inner diameter of 30 mm and an outer diameter of 72 mm. Combined with Equation (15), the calculated *F_BPFI_* values for the three speed conditions are 48.82 Hz, 151.69 Hz, and 255.49 Hz, respectively, while the *F_BPFO_* values are 33.31 Hz, 99.99 Hz, and 112.65 Hz, respectively.

**Figure 4:**
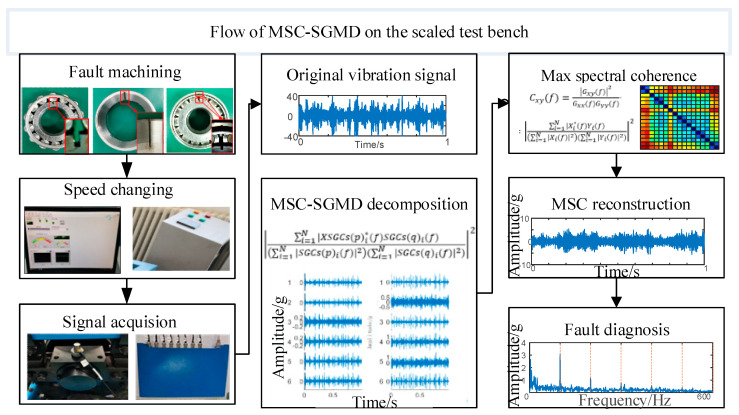
Flow of signal processing of MSC–SGMD.

In order to illustrate the proposed algorithm and its validation effect on the scaled test bench more clearly, the diagnostic flowchart is shown in Figure 4.

### 3.2. Case 1: Inner Race Fault

**Figure 5:**
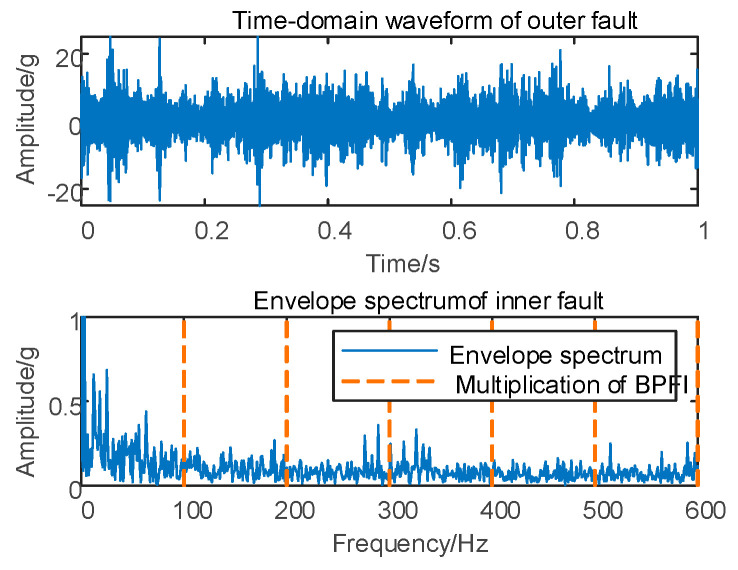
Depiction of 10 km/h time–domain waveform and its envelope spectrum.

In Case 1, fault simulation is conducted, and the vertical vibration signal of the shaft case is acquired and analyzed using the envelope spectrum. The original time domain waveform and envelope spectrum are presented in Figure 5.

As depicted in Figure 5, conducting the envelope spectrum directly on the original signal does not allow for the extraction of the effective fault eigenfrequency. Therefore, it is essential to decompose the original vibration signal based on the proposed MSC–SGMD algorithm.

**Figure 6:**
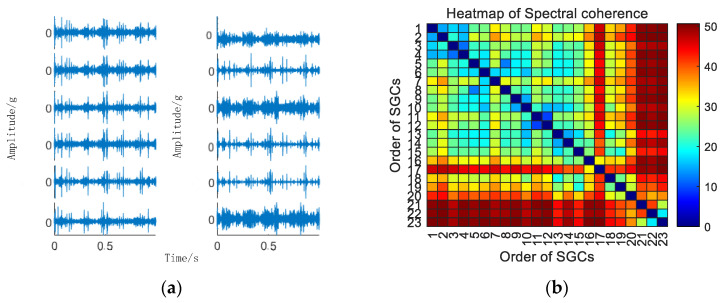
(**a**) 10 km/h MSC–SGSs. (**b**) 10 km/h spectral coherence matrix.

Initially, the SGMD decomposition of the original signal is executed to acquire the corresponding multi–order SGCs components, and the results are illustrated in Figure 6a. Subsequently, the spectral coherence coefficients between individual SGCs are iteratively calculated and characterized in matrix form, and the results of this characterization are presented in Figure 6b.

**Figure 7:**
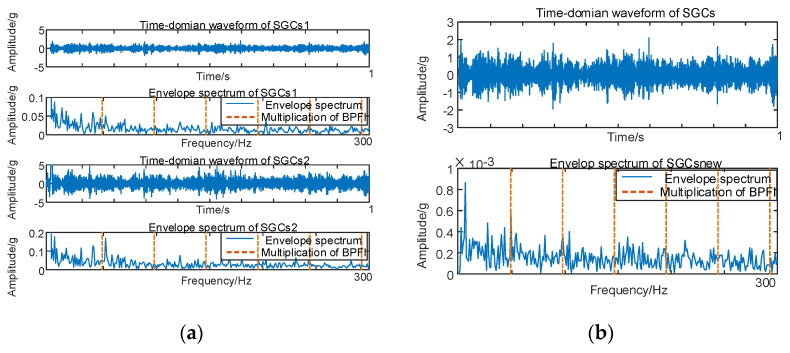
(**a**) Inner race fault for 10 km/h NMSE–SGMD envelope spectrum; (**b**) Inner race fault for 10 km/h MSC–SGMD envelope spectrum.

As illustrated in Figure 6b, there exist variations in spectral coherence coefficients among individual SGCs. For comparative purposes with the conventional NMSE–SGMD algorithm, an analysis is performed on the reconstructed signal envelope spectra of the SGCs within the NMSE–SGMD signal. This analysis is then juxtaposed with the envelope spectra of the MSC–SGMD reconstructed signals. The outcomes of these analyses are presented in Figure 7.

The results in Figure 7 reveal that the conventional NMSE–SGMD identified two SGCs components, both of which were unable to detect *F_BPFI_* and its multiplicative frequency in their envelope spectra. In contrast, the envelope spectrum of the signal reconstructed by MSC–SGMD unmistakably exhibits *F_BPFI_* and its higher–order multiplier frequencies. This substantiates the efficacy and superiority of the proposed algorithm in addressing the 10 km/h inner–race fault.

**Figure 8:**
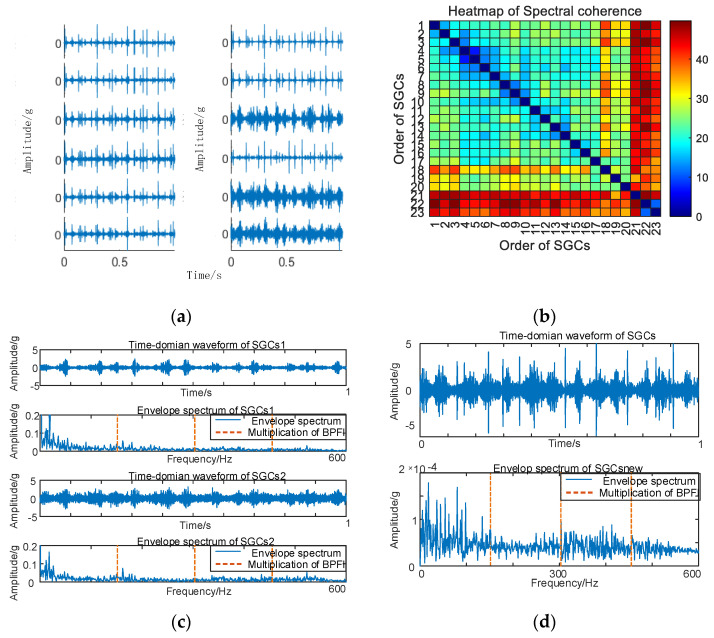
(**a**) Inner race fault for of 30 km/h MSC–SGSs; (**b**) Inner race fault for 0 km/h spectral coherence matrix; (**c**) Inner race fault for30 km/h NMSE–SGMD envelope spectrum; (**d**) Inner race fault for 30 km/h MSC–SGMD envelope spectrum.

Similarly, vibration signal acquisition and MSC–SGMD processing for 30 km/h inner race fault is carried out and the results are shown in Figure 8.

**Figure 9:**
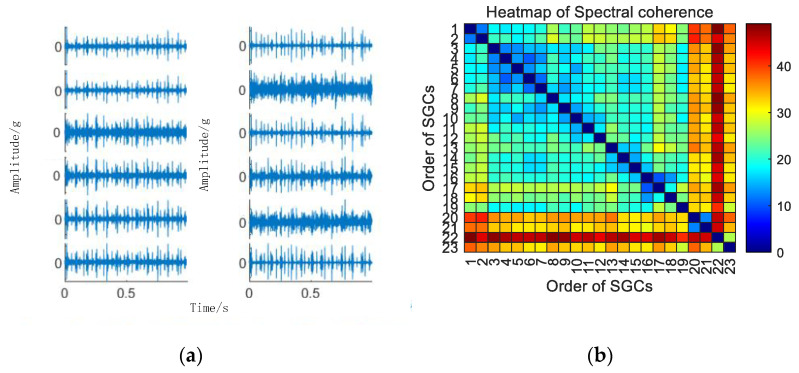

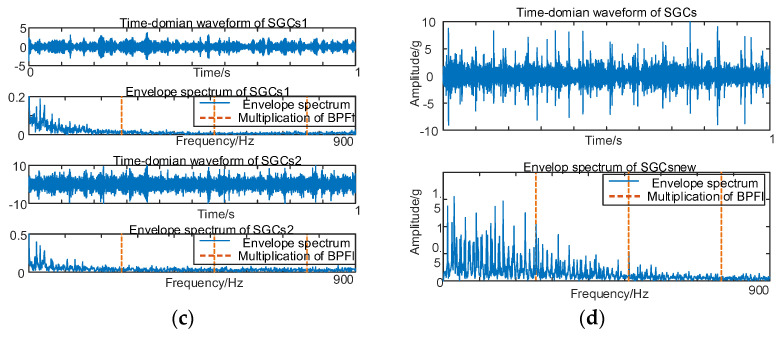
(**a**) Time domain waveform of 50 km/h MSC–SGSs; (**b**) 50 km/h spectral coherence matrix; (**c**) 50 km/h NMSE–SGMD envelope spectrum; (**d**) 50 km/h MSC–SGMD envelope spectrum.

Similarly, vibration signal acquisition and MSC–SGMD processing are conducted for the 50 km/h inner ring failure, and the results are presented in Figure 9.

### 3.3. Case 2: Outer Race Fault

**Figure 10:**
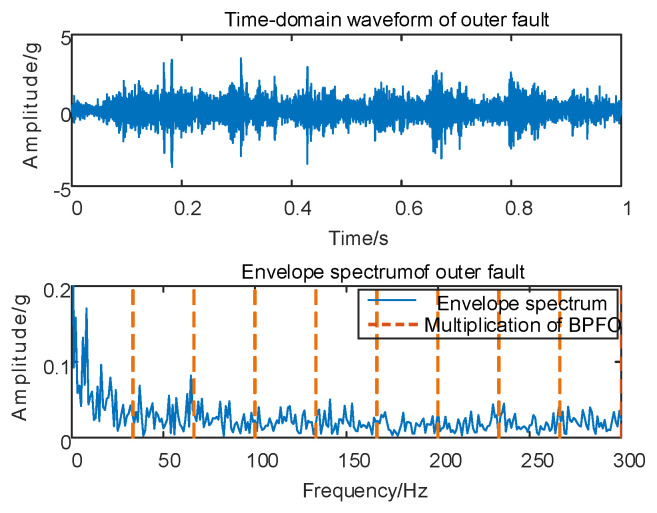
Outer race fault for 10 km/h time domain waveform and its envelope spectrum.

Fault simulation is conducted for Case 2, involving vertical vibration signal acquisition, followed by envelope spectrum analysis on the original vibration signal. The corresponding time domain waveform and envelope spectrum are depicted in Figure 10.

**Figure 11:**
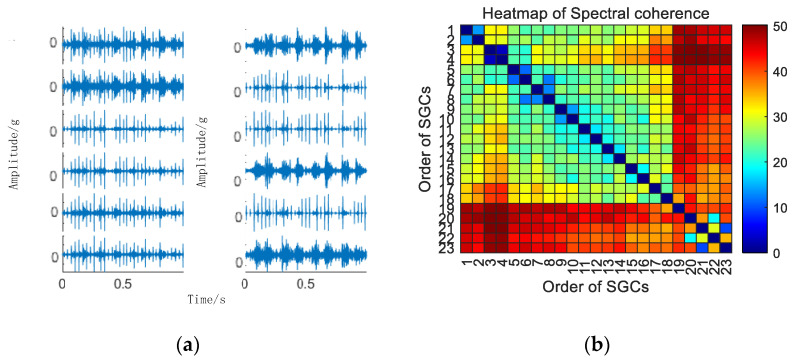
(**a**) Outer race fault for 10 km/h MSC–SGSs; (**b**) 10 km/h spectral coherence matrix.

As depicted in Figure 10, conducting envelope spectrum analysis directly on the original signal proves to be ineffective in extracting fault eigenfrequencies, and the SGMD of the original signal is executed to derive the corresponding multi–order SGCs components, as illustrated in Figure 11a. Subsequently, the spectral coherence coefficients are iteratively computed and matrix–characterized, with the results presented in Figure 11b.

**Figure 12:**
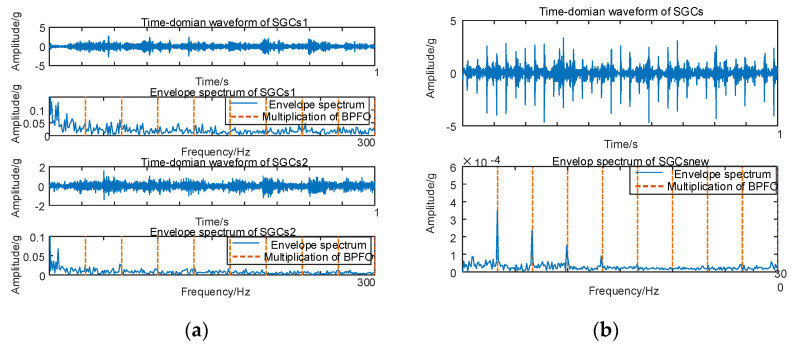
(**a**) Outer race fault for 10 km/h NMSE–SGMD envelope spectrum; (**b**) 10 km/h MSC–SGMD envelope spectrum.

As depicted in Figure 11b, variations exist in the spectral coherence coefficients between individual SGCs, where the MSC components are extracted and reconstructively combined. Subsequently, an analysis is conducted on the envelope spectra of the newly reconstructed signals. For comparative analysis with the conventional NMSE–SGMD algorithm, the envelope spectra of the SGCs within the NMSE–SGMD signals are also scrutinized and contrasted with the MSC–SGMD reconstructed signal envelope spectra. The outcomes of these analyses are presented in Figure 12.

The outcomes depicted in Figure 11 reveal that the conventional NMSE–SGMD identified two SGCs components, both of which were unable to detect *F_BPFO_* and its multiplicative frequency in their envelope spectra. Conversely, *F_BPFO_* and its higher–order multiplier frequencies distinctly manifest in the envelope spectrum of the signal reconstructed by MSC–SGMD. This substantiates the efficacy and superiority of the proposed algorithm in addressing the 10 km/h outer race fault.

**Figure 13:**
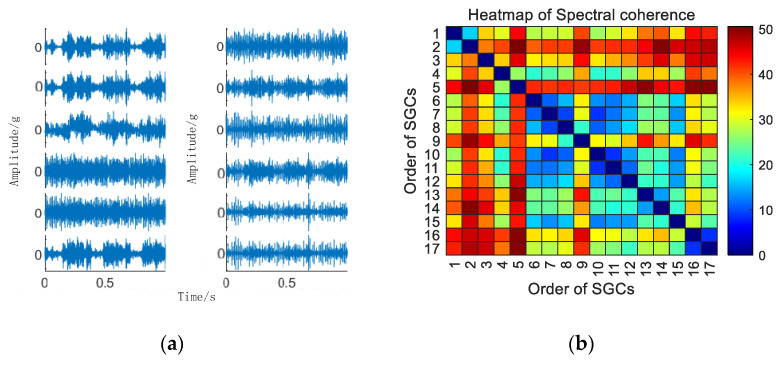

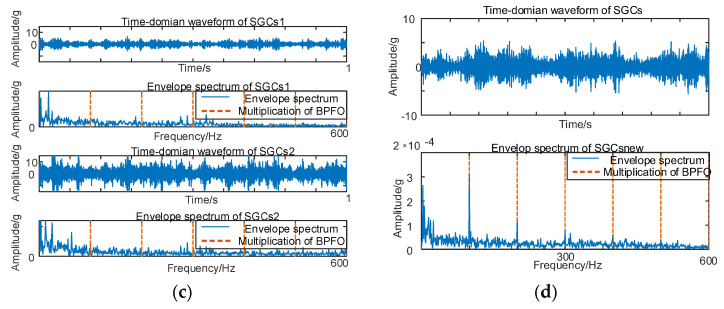
(**a**) Outer race fault for 30 km/h MSC–SGSs; (**b**) 30 km/h spectral coherence matrix; (**c**) 30 km/h NMSE–SGMD envelope spectrum; (**d**) 30 km/h MSC–SGMD envelope spectrum.

Similarly, vibration signal acquisition and MSC–SGMD processing are conducted for the 30 km/h outer race fault, and the results are presented in Figure 13.

**Figure 14:**
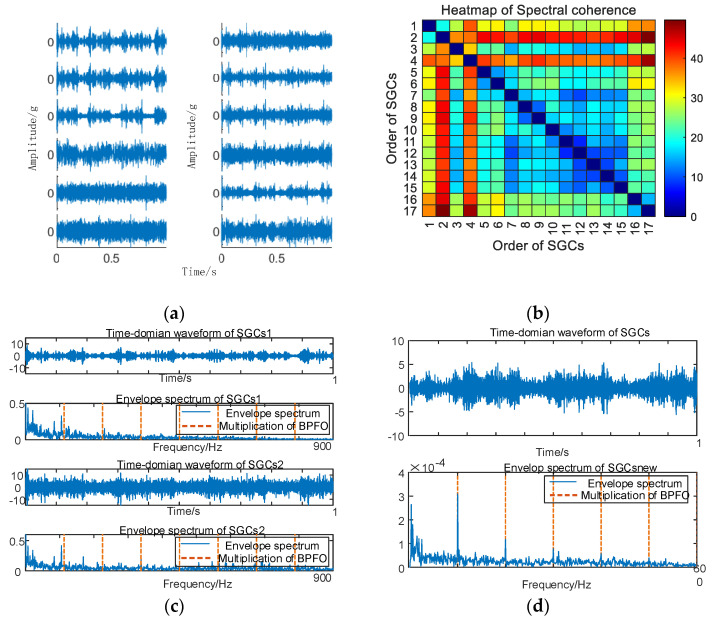
(**a**) Outer race fault for 50 km/h MSC–SGSs; (**b**) 50 km/h spectral coherence matrix; (**c**) 50 km/h NMSE–SGMD envelope spectrum; (**d**) 50 km/h MSC–SGMD envelope spectrum.

Similarly, vibration signal acquisition and MSC–SGMD processing are conducted for the 50 km/h outer race fault, and the results are presented in Figure 14.

### 3.4. Case 3: Compound Fault

**Figure 15:**
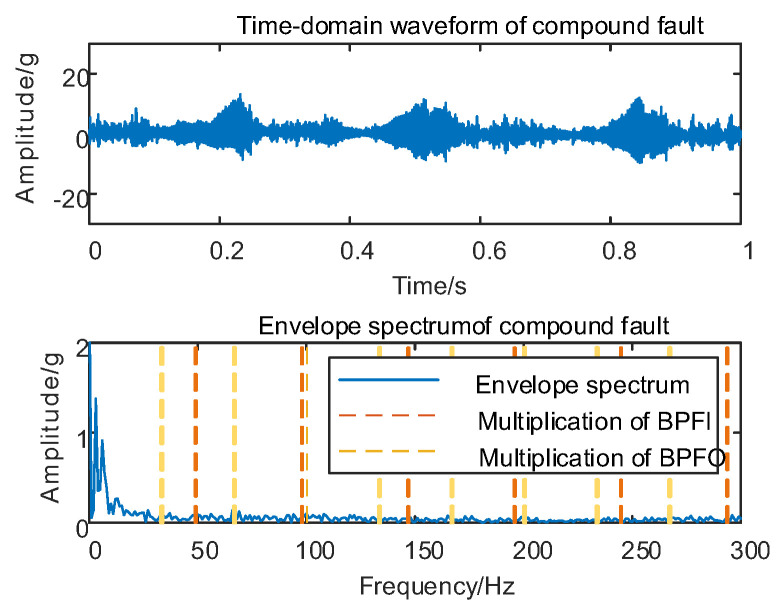
Compound fault for 10 km/h time domain waveform and its envelope spectrum.

Following the validation of the efficacy of the proposed MSC–SGMD algorithm for individual faults in train axlebox bearings, additional tests are conducted to assess vibration conditions. These tests involve the combination of axlebox bearing inner and outer race compound faults at different speeds. The resulting time domain waveforms of axial vertical vibration signals and their respective envelope spectra are presented in Figure 15.

As depicted in Figure 15, conducting envelope spectrum analysis directly on the original signal of the composite fault in the axlebox bearing proved to be ineffective in extracting the effective fault eigenfrequency. Consequently, it becomes imperative to decompose the original vibration signal using the proposed MSC–SGMD algorithm.

**Figure 16:**
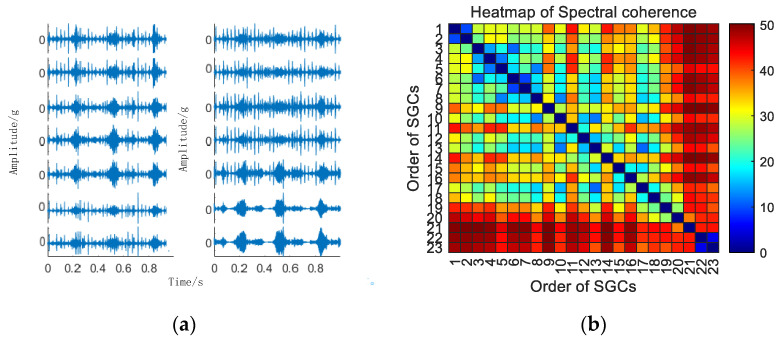
(**a**) Compound fault for 10 km/h MSC–SGSs; (**b**) 10 km/h spectral coherence matrix.

Initially, the SGMD decomposition of the original compound fault vibration signal is executed to derive the corresponding multi–order SGCs components, as illustrated in Figure 16a. Subsequently, the spectral coherence coefficients between individual SGCs are iteratively computed and matrix–characterized, with the results presented in Figure 16b.

**Figure 17:**
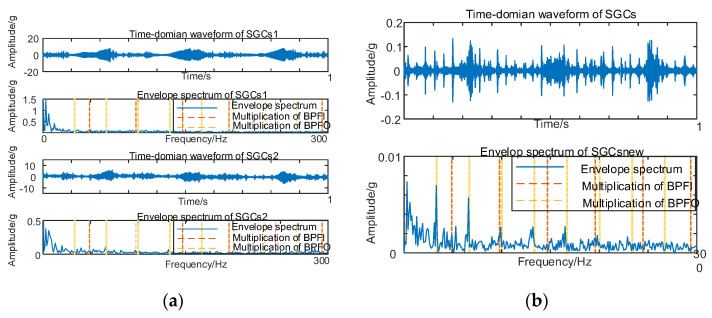
(**a**) Compound fault for 10 km/h NMSE–SGMD envelope spectrum; (**b**) 10 km/h MSC–SGMD envelope spectrum.

Subsequently, the SGC components, identified by the maximum spectral coherence coefficients, are extracted and reconstructed additively. For comparative analysis with the conventional NMSE–SGMD algorithm, an examination is conducted on the reconstructed signal envelope spectra of the SGCs within the NMSE–SGMD signals. In order to compare with the conventional NMSE–SGMD algorithm, the reconstructed signal envelope spectra of the SGCs of the NMSE–SGMD signals are also analyzed and compared to the MSC–SGMD reconstructed signal envelope spectra. The results are shown in Figure 17.

**Figure 18:**
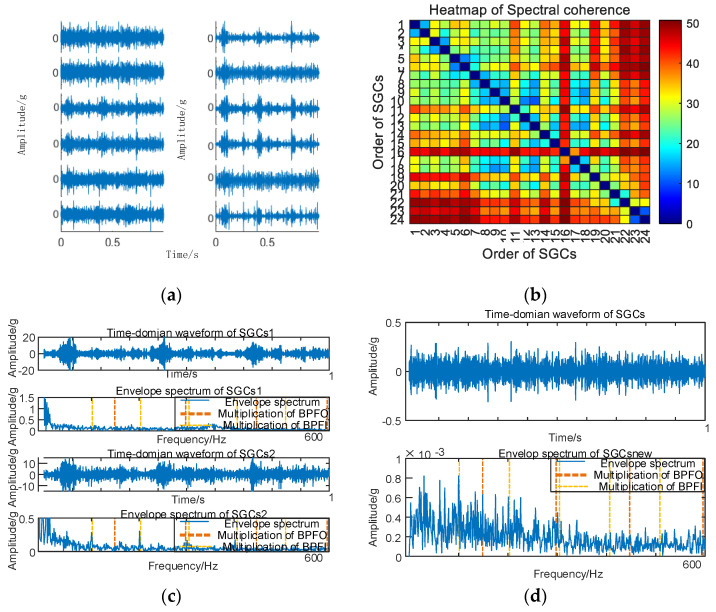
(**a**) Compound fault for 30 km/h MSC–SGSs; (**b**) 30 km/h spectral coherence matrix; (**c**) 30 km/h NMSE–SGMD envelope spectrum; (**d**) 30 km/h MSC–SGMD envelope spectrum.

The envelope spectrum of the signal decomposed and reconstructed by the MSC–SGMD algorithm, as shown in Figure 17b, distinctly exhibits the fundamental frequency of *F_BPFO_* along with *F_BPFI_* and its multiplicative frequency. In contrast, the results obtained from the traditional NMSE–SGMD algorithm fail to reveal these corresponding components. This disparity underscores the effectiveness and superiority of the proposed algorithm. Similarly, vibration signal acquisition and MSC–SGMD processing are conducted for a 30 km/h compound fault, and the results are presented in Figure 18.

**Figure 19:**
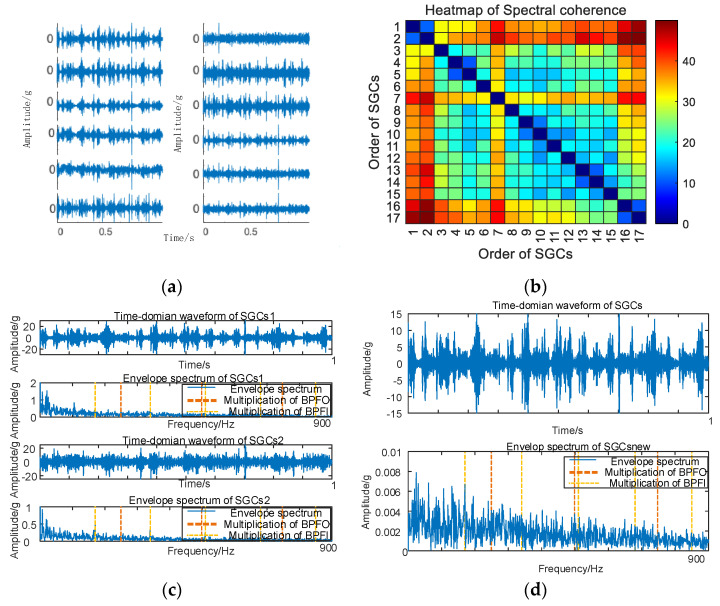
(**a**) Compound fault for 50 km/h MSC–SGSs; (**b**) 50 km/h spectral coherence matrix; (**c**) 50 km/h NMSE–SGMD envelope spectrum; (**d**) 50 km/h MSC–SGMD envelope spectrum.

Similarly, the vibration signal acquisition and MSC–SGMD processing for the 50 km/h compound fault is carried out, and the results are shown in Figure 19.

## 4. Discussion

(1) In the present study, scholars have proposed many methods for extracting the fault characteristics of axle box bearings, such as the SGMD algorithm, etc. However, these algorithms have problems such as poor extraction effect and submerged fault feature frequency when diagnosing the compound fault of axle box bearing. By incorporating the concept of maximum spectral coherence into the single–component reconstruction of SGCs, we effectively extract spectral discreteness between components. This method is applied to reconstruct vibration signals related to bearing faults. Furthermore, we process and collect acceleration signals from axle box bearings across different speeds on an equal scale test rig for algorithm validation.
(2) In contrast to traditional methods, the proposed multi–scale correlation sparse grouping mode decomposition (MSC–SGMD) algorithm exhibits enhanced capability in decomposing and reconstructing fault–induced vibration signals of axle box bearings. This effectiveness is demonstrated under various fault conditions, including inner ring, outer ring, and compound faults. The algorithm successfully identifies the associated fault characteristic frequencies and high–order frequency doubling. In comparison, the conventional normalized mean square error sparse grouping mode decomposition (NMSE–SGMD) algorithm falls short in effectively extracting harmonic frequency components. Notably, under compound fault scenarios, the traditional SGMD algorithm proves inadequate in extracting the pertinent fault characteristic frequencies at speeds of 10 km/h, 30 km/h, and 50 km/h.
(3) The validation results indicate that at speeds of 10 km/h, 30 km/h, and 50 km/h, the presence of additional components—along with a pronounced hunting motion influence—results in a more conspicuous amplitude modulation in the signals, particularly in the form of frequency modulation. This modulation causes the characteristic frequency of bearing faults to be submerged within redundant components. As a consequence, traditional vertical vibration signal envelope spectrum analysis proves inadequate for fault diagnosis. Meanwhile, the NMSE–SGMD algorithm, exclusively reconstructing signals for time domain correlation, produces two reconstructed signals corresponding to different speeds and fault conditions. However, the envelope spectral analysis of these reconstructed signals fails to distinctly highlight the characteristic frequency of bearing faults.
(4) While the proposed algorithm exhibits commendable fault feature extraction capability, experimental verification results reveal that as the speed increases, the fault feature frequency in the decomposed vibration signal by MSC–SGMD becomes less conspicuous and is partially obscured by redundant components. This phenomenon may stem from the fact that as speed escalates, the amplitude growth of the fault impact on the axle box bearing lags behind the rate of increase in vibration noise. Consequently, there is a discernible reduction in the signal–to–noise ratio, resulting in a corresponding decline in the feature extraction effectiveness of the proposed algorithm.
(5) In future research endeavors, the feature extraction capability of the proposed MSC–SGMD under elevated noise conditions can be validated through augmenting the linear speed of the wheelset, intensifying the dynamic vertical load, and exploring other operational scenarios. Furthermore, optimization of the calculation mode for the maximum spectral coherence coefficient can enhance the algorithm’s accuracy and practical applicability.

## 5. Conclusions

This paper asserts the theoretical significance and experimental validation of employing the maximum spectral coherence for diagnosing faults in axle box bearings, with a particular emphasis on the extraction of features related to compound faults. Through this investigation, we aim to enhance the precision and applicability of the traditional SGMD method, offering an effective and accurate algorithm for extracting fault features in the diagnosis of train axle box bearing faults. By introducing the spectral coherence coefficient to characterize the spectral coherence between independent components, the proposed algorithm achieves accurate reconstruction of key components, effectively suppressing noise and extracting main fault features. Furthermore, experimental verification under varying speed conditions successfully extracts fault characteristics, enabling the effective diagnosis of compound faults in the axle box bearing. However, this study is constrained by the influence of wheel set speed and load difficulty, making it challenging to validate diagnostic efficacy under broader speed ranges and higher noise levels. In future investigations, escalating the speed levels and amplifying noise impact will allow a more robust verification of the proposed method’s superiority in diagnosing axle box bearing faults.

## Figures and Tables

**Table 1:** Bearing fault conditions.

Case Type	Speed 1	Speed 2	Speed 3
Case 1 (Inner race fault)	10 km/h	30 km/h	50 km/h
Case 2 (Outer race fault)	10 km/h	30 km/h	50 km/h
Case 3 (Compound fault)	10 km/h	30 km/h	50 km/h

## Data Availability

Data sharing is not applicable to this article as no datasets were generated during the current study.
